# Supplementary material for: Neonatal Vitamin C and Cysteine Deficiencies Program Adult Hepatic Glutathione and Specific Activities of Glucokinase, Phosphofructokinase, and Acetyl-CoA Carboxylase in Guinea Pigs’ Livers
Source: Antioxidants (Basel). 2021 Jun 12;10(6):953. doi: 10.3390/antiox10060953 (PMC8231532; doi:10.3390/antiox10060953)
Supplement: Supplementary file 1 [file antioxidants-10-00953-s001.zip › antioxidants-1241111-supplementary.pdf]

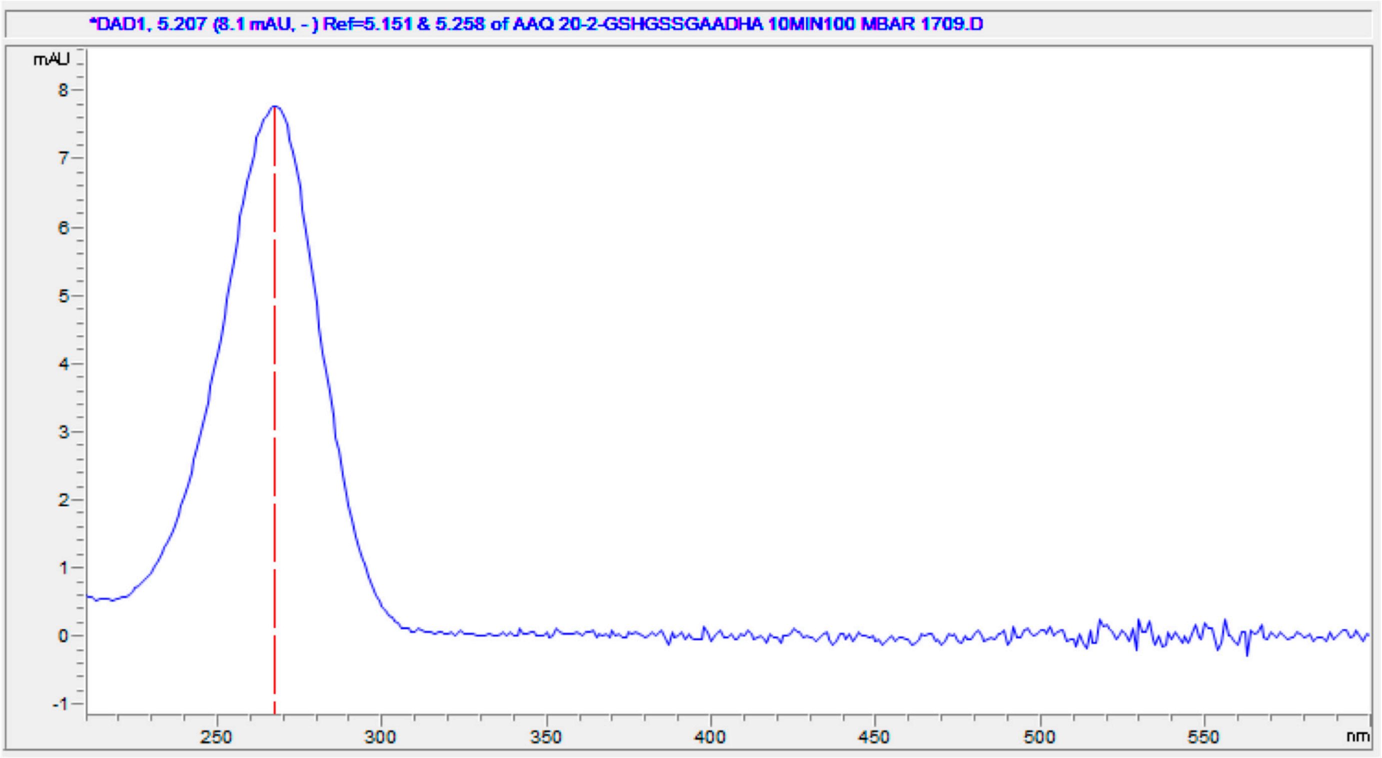

Supplemental Figure 1. Absorbance maxima of a solution of ascorbate 20 µM in metaphosphoric acid 1%, resolved in boric acid 200 mM, acetonitrile 20%, pH 9.6 at 268 nm in capillary electrophoresis.

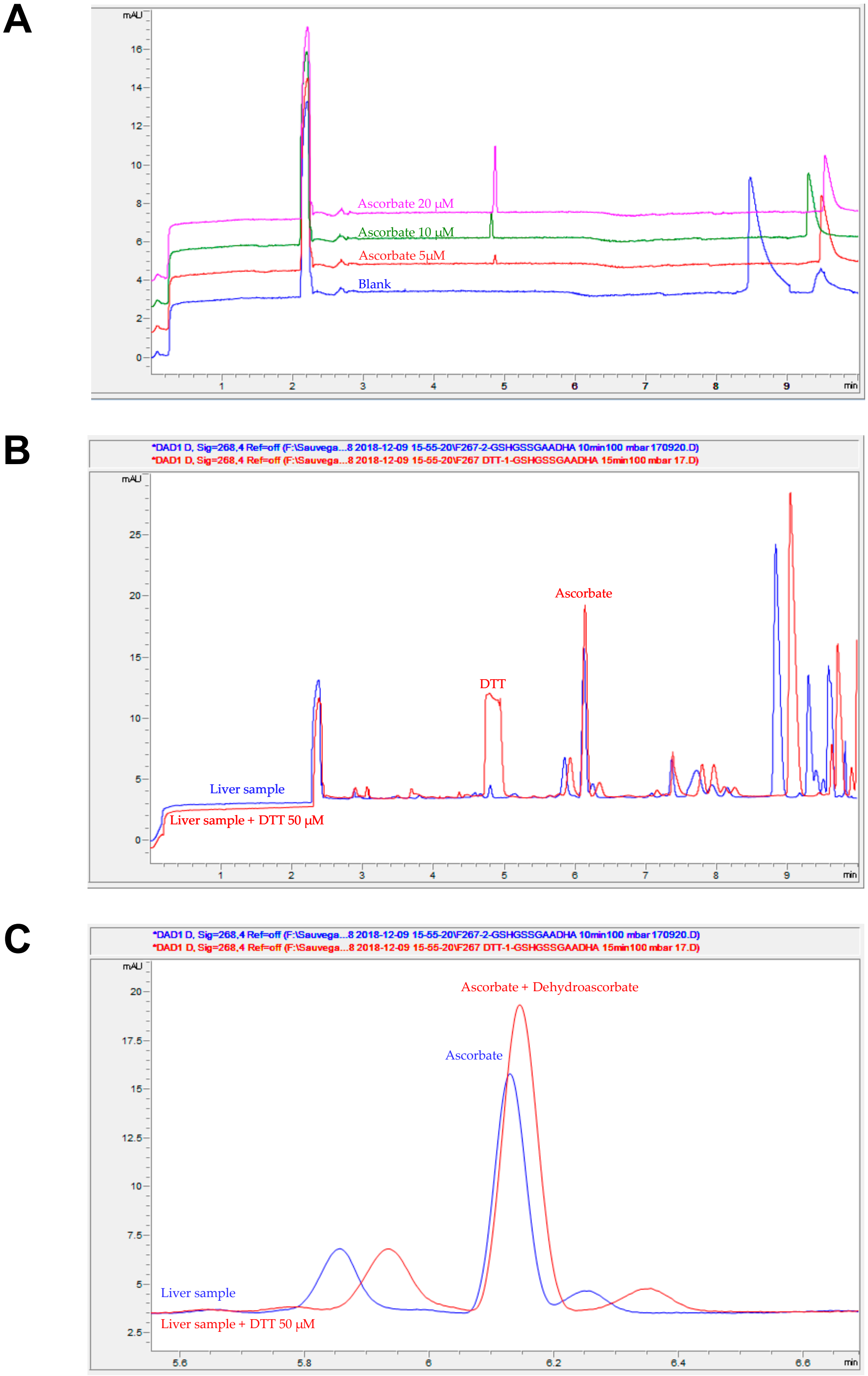

Supplemental Figure 2. Quantification of ascorbate and dehydroascorbate by capillary electrophoresis. (A): Chromatoherograms of the standard curve of ascorbate, depicting the absence of contaminating peaks; (B): Shift in peaks caused by the addition of reducing agent DTT. (C): Close-up at the region of interest of ascorbate from figure 2B that shows the increase in ascorbate peak area due to reduction of dehydroascorbate into ascorbate.

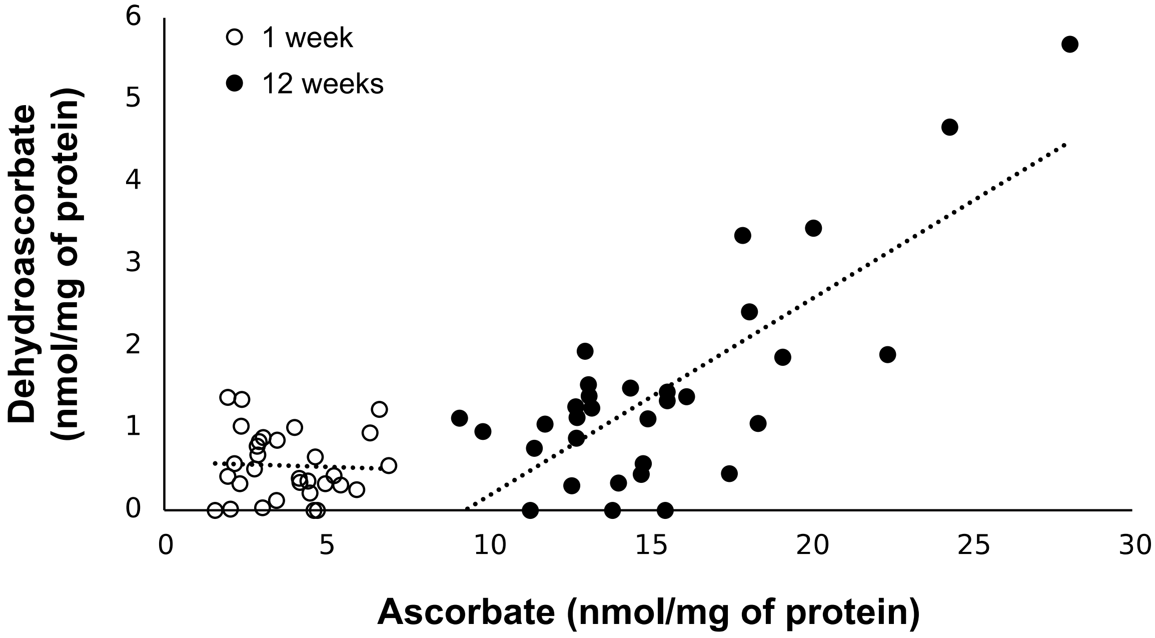

Supplemental Figure 3. Correlation between liver ascorbate and dehydroascorbate levels in 1-week and 12-week animals. The correlation was non-significant at 1 week of age (DHA = (-0.0115 • AA) + 0.5881; $r^2 = 0.0018$), but significant at 12 weeks of age (DHA = (0.2395 • AA) - 2.2368; $r^2 = 0.5902$; $p<0.001$).
